# Association Between History of Gestational Diabetes Mellitus and the Risk of Urinary Leakage in Women: A Cross‐Sectional Study

**DOI:** 10.1002/hsr2.71243

**Published:** 2025-09-22

**Authors:** Huiwen Hu, Bin Yu, Mei Xiang, Ziyi Guo, Huihui Wang, Li Wang

**Affiliations:** ^1^ Changzhou Maternal and Child Health Care Hospital, Changzhou Medical Center Nanjing Medical University Changzhou Jiangsu Province China

**Keywords:** BMI, GDM, SII, UL, WBC

## Abstract

**Background and Aims:**

Gestational diabetes mellitus (GDM) and urinary leakage (UL) are common health issues affecting women. While certain adverse effects of GDM are known risk factors for UL, the direct association between GDM and UL remains underexplored. This study aims to investigate this relationship.

**Methods:**

Data from 13,417 women in the National Health and Nutrition Examination Survey (NHANES) (2007–2020) were analyzed. Multivariate logistic regression models were used to explore the association between GDM and UL. Stratified and subgroup analyses, adjusted for confounding factors, and mediation analysis were conducted to investigate potential mediators.

**Results:**

GDM is associated with an increased risk of UL. According to the multivariate logistic regression model, the unadjusted analysis showed that women with a history of GDM faced a 20.0% higher risk of UL (OR = 1.20, 95% CI 1.05–1.37, *p* < 0.01). After adjusting for confounding factors such as age, body mass index (BMI), and white blood cell (WBC) counts, the association remained robust, with an OR of 1.32 (95% CI 1.16–1.53, *p* < 0.001). This association was especially pronounced among women who smoked, were aged below 30 or above 35 years, had a poverty ratio less than 5, and exhibited WBC counts below 1000 cells/μL. Additionally, age, BMI, WBC, and systemic inflammation index (SII) were all positively linked to the severity of UL, with older age, higher BMI, greater WBC counts, and elevated SII levels corresponding to more severe UL in women. Mediation analysis revealed that both BMI and WBC count partially mediated the relationship between GDM and UL. Furthermore, after covariate adjustment, a nonlinear positive relationship was observed, with the inflection point for BMI occurring at 34.88.

**Conclusions:**

Based on our results, we conclude that women diagnosed with GDM elevate the risk of UL, and BMI, WBC count appear to serve as mediators in this association.

**Abbreviations**
BMI: body mass index
CBC: complete blood count
CI: confidence intervals
GDM: gestational diabetes mellitus
NHANES: National Health and Nutrition Examination Survey
OR: odds ratios
SII: systemic immune‐inflammation index
UL: urinary leakage
WBC: white blood cell

## 1 Introduction

Gestational diabetes mellitus (GDM) represents a significant public health challenge, with a pooled global standardized prevalence of 14.0% [1, 2]. The incidence of GDM continues to climb, driven by the growing number of obese women of childbearing age and the increasing age at which women give birth [3]. GDM poses substantial long‐term health risks for both pregnant women and their offspring, including metabolic disorders, postpartum obesity, macrosomia, and cardiovascular diseases [4, 5, 6]. White blood cell (WBC) count, an indicator of the body's inflammatory state, has been shown to play a critical role in GDM [7]. Elevated WBC levels signal a heightened inflammatory response, which is associated with a greater risk of complications for pregnant women. Thus, monitoring and managing WBC counts can be a valuable strategy for the early detection and prevention of GDM‐related complications.

A common symptom of female pelvic floor dysfunction, affecting millions of women each year [8]. Among postpartum women, approximately 33% were reported to have experienced urinary leakage (UL), which severely impacts their quality of life and poses a significant economic and healthcare burden on the country [9, 10, 11]. It is well‐established that pregnancy and childbirth are independent risk factors for female pelvic floor dysfunction, with prolonged second‐stage labor and macrosomia identified as major contributors [12, 13, 14, 15]. While some long‐term complications of GDM, such as weight and age, are recognized risk factors for UL, epidemiological studies have yet to clarify the relationship between a history of GDM and the risk of UL in women, leaving the key factors driving this association uncertain.

Based on these observations, we hypothesize that a correlation exists between GDM and UL. By leveraging data mining from NHANES 2007–2020, we aim to elucidate the association between a history of GDM and the risk of UL in women, with a particular emphasis on identifying the mediating factors involved.

## 2 Materials and Methods

### 2.1 Study Design and Participants

NHANES (National Health and Nutrition Examination Survey) is a research program aimed at assessing the health and nutritional status of American adults and children, with a database that provides various health and nutrition‐related information about the participants. The study data is based on information related to the history of gestational diabetes from the NHANES database from 2007 to 2020. This study was reported in accordance with the STROBE guidelines for cross‐sectional studies.

**Figure 1:**
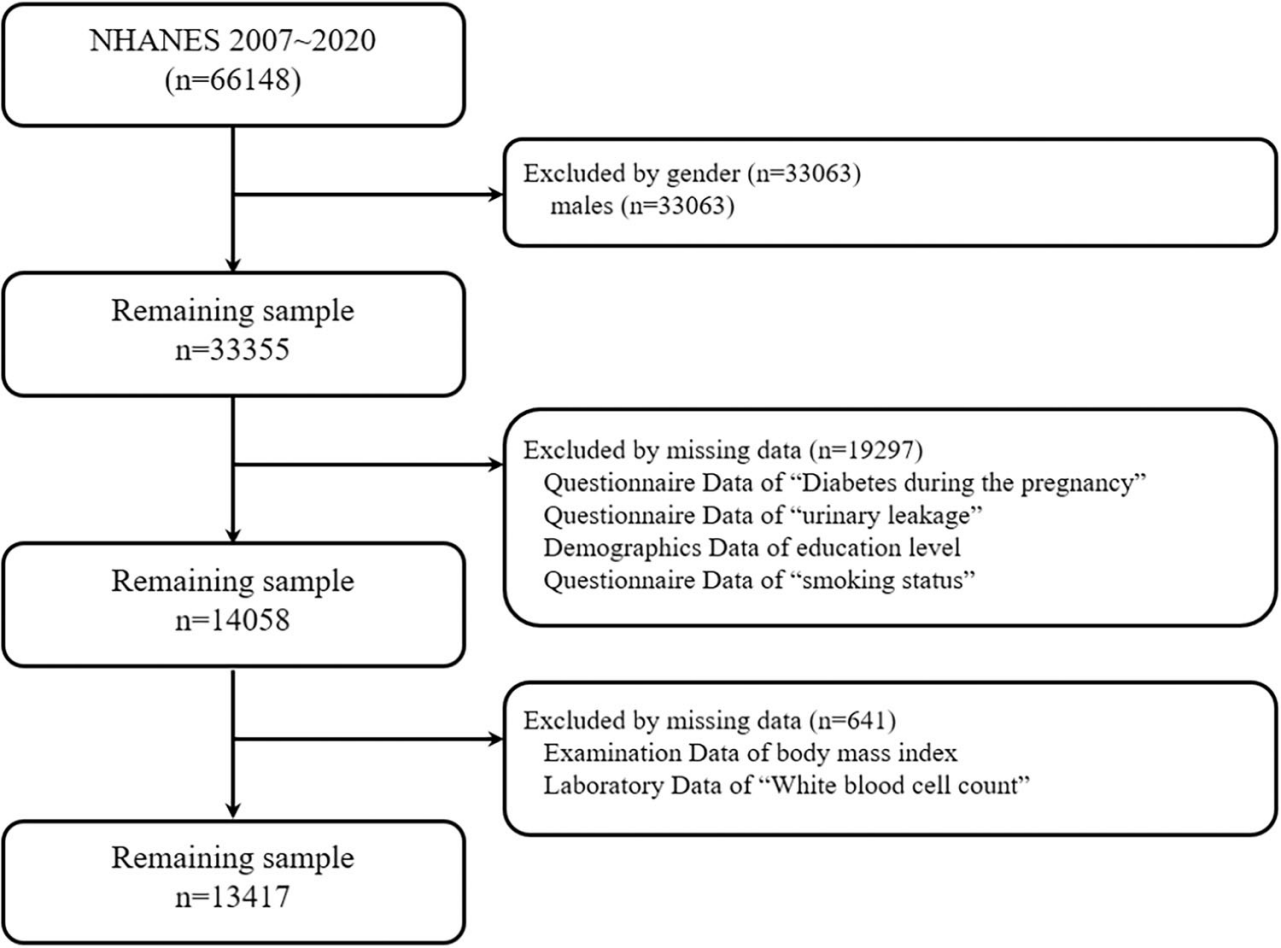
Flowchart of participants selection from the NHANES 2007–2020.

The inclusion criteria are as follows: (1) female; (2) answered questions regarding the history of GDM; (3) answered questions about UL. Participants with missing information on GDM and UL, as well as participants with missing covariates (education level, smoking status, BMI, WBC), were also excluded. Ultimately, a total of 13,417 cases were included in this study. The detailed selection process is illustrated in Figure 1.

#### 2.1.1 Gestational Diabetes Mellitus

GDM history was identified from the Survey questionnaire (RHQ162): During your pregnancy, were you ever told by a doctor or other health professional that you had diabetes, sugar diabetes, or gestational diabetes? Please do not include diabetes that you may have known about before the pregnancy. When the answer is “Yes”, it is considered a history of gestational diabetes (study subjects).

#### 2.1.2 Urinary Leakage

To assess UL, we utilized the following question (KIQ005, KIQ042, KIQ044, KIQ046), any woman who answered “yes”, it was considered to have the history of UL. For example, when asked “How often have you experienced urinary leakage? (KIQ005)”, a response of “never” was considered as no urinary leakage, while any other response was considered as having UL, and they were grouped according to their responses: UL 1: Less than once a month. UL 2: A few times a month. UL 3: A few times a week. UL 4: Every day and/or night.

#### 2.1.3 Main Variables

The primary independent variable, systemic immune‐inflammation index (SII), is a novel inflammation marker calculated by multiplying platelet and neutrophil counts and then dividing by the lymphocyte count. To derive the SII, data from NHANES's “Complete Blood Count (CBC)” segments were utilized, specifically employing lymphocyte (LBDLYMNO), neutrophil (LBDNENO), and platelet (LBXPLTSI) counts. These values were then used to calculate the SII for each participant, which was further categorized into four questionnaires (Q1–Q4) for analysis. Q1 (SII < 249), Q2 (249~604.55), Q3 (604.55~825.35), and Q4 (SII > 825.35).

BMI was identified from the Examination Data “BMXBMI—Body Mass Index (kg/m^2^)”, and the levels were divided as follows: under/normal weight: < 25 kg/m^2^, overweight: 25 to < 30 kg/m^2^, obesity: > 30 kg/m^2^.

#### 2.1.4 Other Covariates

Referring to the published research results, the following covariates were included: age, race, poverty income ratio, education level, and smoking status.

### 2.2 Statistical Analysis

Baseline tables were constructed utilizing the DecisionLinnc V1.0 (DecisionLinnc Core Team, 2023. DecisionLinnc. 1.0. https://www.statsape.com/) by grouping participants with and without GDM according to the characteristics of the overall cohort. Sample size and proportion (*n* (%)) were used to present categorical variables, while mean and standard deviation (mean (SD)) were utilized to present continuous variables (*n*: unweighted; *n* (%), mean and SD: weighted). To investigate the association of GDM with UL, we employed multivariable logistic regression models, which estimate odds ratios (OR) and 95% confidence intervals (CI) to assess the strength of associations between variables. This analysis was pre‐specified as the primary method to evaluate the GDM‐UL relationship. Subgroup analysis of associations between a history of GDM and UL was performed as an exploratory analysis to account for potential confounding variables, such as age, smoking status, and BMI, with interaction terms (*P*‐interaction) tested for effect modification. Mediation analysis was conducted as an exploratory analysis to examine the mediating roles of BMI and WBC count in the association between GDM and UL [16]. All analyses were performed with a pre‐specified significance level of *α* = 0.05, using two‐sided tests unless otherwise specified. Statistical analyses, including logistic regression, subgroup analysis, and mediation analysis, were conducted using R version 4.2.1 (R Core Team, 2022), and reporting adhered to SAMPL guidelines.

## 3 Results

### 3.1 Baseline Characteristics of Participants

A total of 13,417 participants were enrolled in this study. Table 1 provides an overview of the baseline characteristics, revealing a GDM prevalence of 7.97%. Significant differences were identified between women with a history of GDM and those without, particularly in variables such as age, race, education level, BMI, blood pressure related indicators (systolic and diastolic blood pressure), and blood cell count‐related indicators (red blood cell count, white blood cell count, neutrophil count, lymphocyte count, and platelet count) (*p* < 0.01). The overall prevalence of UL was 62.70% (8413 individuals). Compared to the group without GDM, the incidence of UL was higher among women with a history of GDM. This finding underscores a definitive association between a history of GDM and the occurrence of UL.

**Table 1:** Baseline participant characteristics according to gestational diabetes mellitus (GDM).

Variable names	Overall	Women with GDM	Women without GDM	*p* value
*n*	13,417	1069	12,348	
Age (year)	52.21 ± 16.61	45.72 ± 12.41	52.77 ± 16.81	< 0.01
Race (%)				
Mexican American	2064 (15.38)	224 (20.95)	1840 (14.90)	< 0.001
Other Hispanic	1583 (11.80)	125 (11.69)	1458 (11.81)	
Non‐Hispanic White	5390 (40.17)	358 (33.49)	5032 (40.75)	
Non‐Hispanic Black	3033 (22.61)	224 (20.95)	2809 (22.75)	
Other race	1347 (10.04)	138 (12.91)	1209 (9.79)	
Poverty income ratio	2.4 ± 1.52	2.34 ± 1.55	2.406 ± 1.52	0.18
Education level (%)				
Less than 9th grade	1399 (10.43)	103 (9.64)	1296 (10.50)	0.01
9–11th grade	1942 (14.47)	150 (14.03)	1792 (14.51)	
High school graduate	3059 (22.80)	205 (19.18)	2854 (23.11)	
Some college	4285 (31.94)	388 (36.30)	3897 (31.56)	
College graduate or above	2732 (20.36)	223 (20.86)	2509 (20.32)	
BMI (kg/m^2^)	30.16 ± 7.63	32.09 ± 7.83	30.00 ± 7.59	< 0.01
Normal weight (%)	3547 (26.44)	186 (17.40)	3361 (27.22)	< 0.001
Overweight (%)	3920 (29.22)	274 (25.63)	3646 (29.53)	
Obesity (%)	5950 (44.35)	609 (56.97)	5341 (43.25)	
Smoking status (%)				
Yes	5030 (37.49)	392 (36.67)	4638 (37.56)	0.59
No	8387 (62.51)	677 (63.33)	7710 (62.44)	
Systolic: Blood pressure (mm Hg)	124.07 ± 19.99	121.27 ± 18.41	124.3 ± 20.1	< 0.01
Diastolic: Blood pressure (mm Hg)	68.88 ± 12.8	70.31 ± 11.99	68.76 ± 12.86	< 0.01
Red blood cell count (million cells/µL)	4.44 ± 0.42	4.47 ± 0.41	4.44 ± 0.42	0.01
White blood cell count (1000 cells/µL)	7.27 ± 2.28	7.7 ± 2.26	7.24 ± 2.28	< 0.01
Neutrophils number (1000 cell/µL)	4.29 ± 1.74	4.57 ± 1.8	4.26 ± 1.73	< 0.01
Lymphocyte number (1000 cells/µL)	2.22 ± 0.97	2.36 ± 0.75	2.21 ± 0.99	< 0.01
Platelet count (1000 cells/µL)	257.47 ± 67.32	266.23 ± 69.04	256.72 ± 67.12	< 0.01
Systemic immune‐inflammation index (SII)	545.77 ± 352.17	558.26 ± 329.56	544.69 ± 354.05	0.23
Q1 (%)	1444 (10.76)	1347 (10.91)	97 (9.07)	0.39
Q2 (%)	7728 (57.60)	7101 (57.51)	627 (58.65)	
Q3 (%)	2385 (17.78)	2198 (17.80)	187 (17.49)	
Q4 (%)	1833 (13.66)	1677 (13.58)	156 (14.59)	
Urinary leakage				
Yes	8413 (62.70)	711 (66.51)	7702 (62.37)	0.01
No	5004 (37.30)	358 (33.49)	4646 (37.63)	

### 3.2 Logistic Regression Analysis

Table 2 delineates the effect sizes, odds ratios (OR), and 95% confidence intervals (CI) derived from three multi‐variable logistic regression models. In Model III, after adjusting for demographic variables such as age, BMI, and WBC counts, it was found that women with a history of GDM was a significant risk of developing UL (OR = 1.32, 95% CI 1.16, 1.53), which exhibited a 32.0% increased risk of developing UL, compared to those without the GDM history. This elevated risk remained statistically significant difference after adjusting for age, race, education level, poverty, smoking status in Model II (OR = 1.47, 95% CI 1.28, 1.68) and Model I (OR = 1.20, 95% CI 1.05, 1.37). Results under these models indicated a statistically significant positive correlation between a history of GDM and UL.

**Table 2:** Multi‐variable‐adjust ORs and 95% CI of history of GDM and UL.

History of GDM	Model 1		Model 2		Model 3	
	OR (95% CI)	*p* value	OR (95% CI)	*p* value	OR (95% CI)	*p* value
No	Reference		Reference		Reference	
Yes	1.20 (1.05, 1.37)	0.007	1.47 (1.28, 1.68)	< 0.001	1.32 (1.16, 1.53)	0.001

*Note:* Model 1 without adjustments.

Model 2 was additionally adjusted for age, race, education level, poverty, and smoking status.

Model 3 was additionally adjusted for BMI and WBC.

### 3.3 Subgroup Analysis

**Figure 2:**
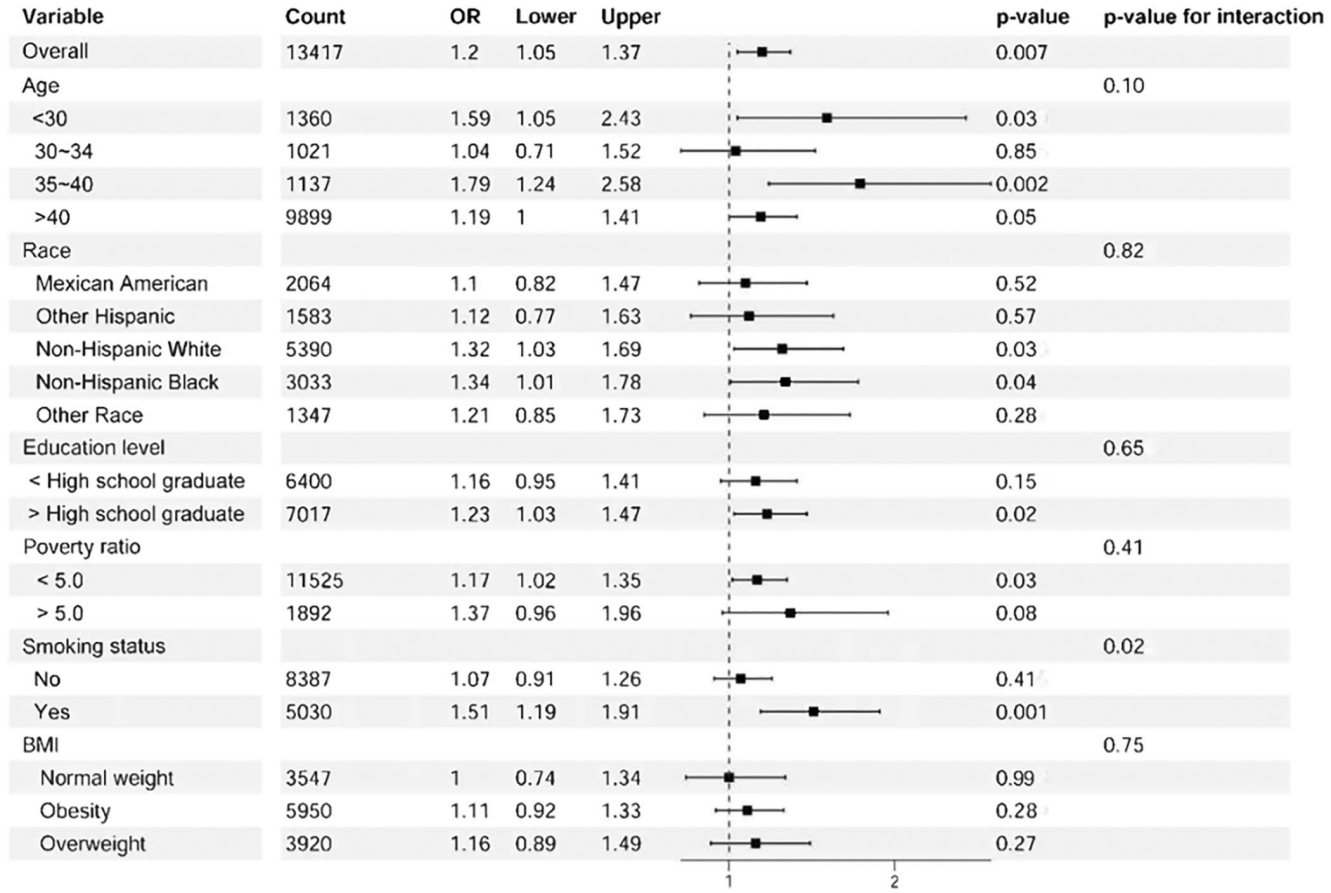
Subgroups analyses of the effect of history of GDM and UL.

Table 3 and Figure 2 present the results of subgroup analyses based on age, poverty income ratio, smoking, BMI, and WBC. The analysis indicated a stronger association between GDM and UL in women who smoked, were younger than 30 or older than 35 years, had a poverty income ratio below 5, or had WBC counts less than 1000 cells/μL. However, with the exception of smoking status (*p*‐interaction = 0.02), no statistically significant effect modification was observed for the association between GDM and UL across the stratification variables.

**Table 3:** Subgroup analysis of associations between history of GDM and UL.

Subgroups	*n*	OR (95% CI)	*p* value	*p* value for interaction
Overall	13,417	1.2 (1.05, 1.37)	0.007	
Age				0.10
< 30	1360	1.59 (1.05, 2.43)	0.03	
30~34	1021	1.04 (0.71, 1.52)	0.85	
35~40	1137	1.79 (1.24, 2.58)	0.002	
> 40	9899	1.19 (1, 1.41)	0.05	
Poverty ratio				0.41
< 5.0	11,525	1.17 (1.02, 1.35)	0.03	
> 5.0	1892	1.37 (0.96, 1.96)	0.08	
Smoking status				0.02
Yes	5030	1.51 (1.19, 1.91)	0.001	
No	8387	1.07 (0.91, 1.26)	0.41	
BMI				0.75
Normal weight	3547	1 (0.74, 1.34)	0.99	
Overweight	3920	1.16 (0.89, 1.49)	0.27	
Obesity	5950	1.11 (0.92, 1.33)	0.28	
WBC				
< 1000 cells/μL	12,038	1.15 (1, 1.33)	0.05	0.27
> 1000 cells/μL	1379	1.45 (0.99, 2.11)	0.05	

### 3.4 Mediation Analysis

**Figure 3:**
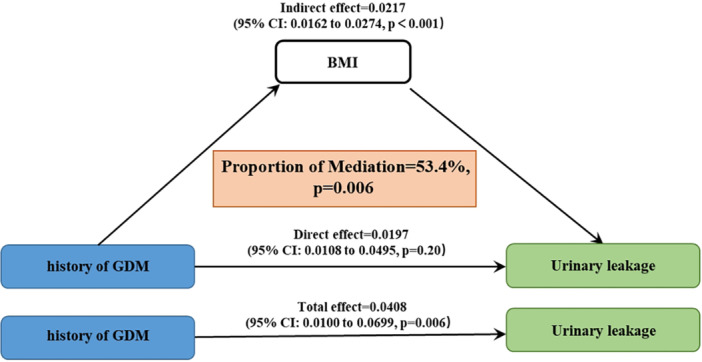
Parallel mediation analysis between GDM and UL by BMI.

**Figure 4:**
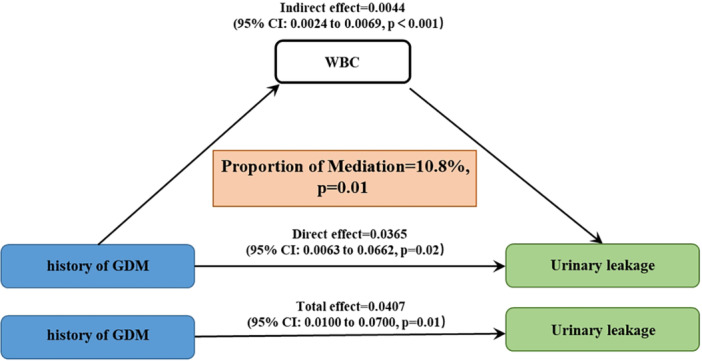
Parallel mediation analysis between GDM and UL by WBC.

**Figure 5:**
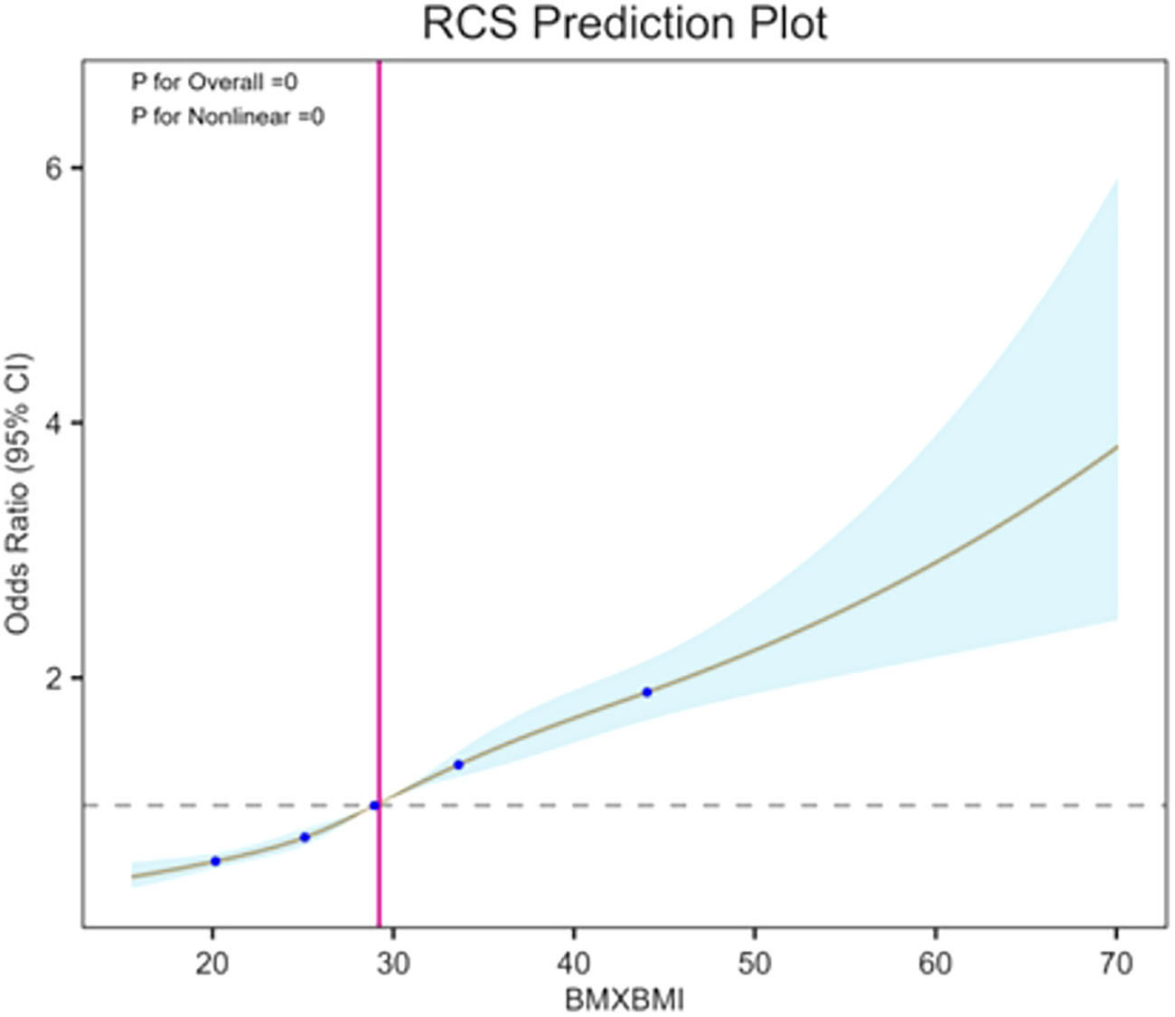
Restricted cubic splines curves for the association between GDM history and UL by increased BMI levels. Results were adjusted for age, race, education level, poverty income ratio, and smoking.

The parallel mediation analysis revealed that BMI and WBC count significantly mediated the relationship between a history of GDM and UL, with moderation ratios of 53.4% and 10.8%, respectively (*p *< 0.01) (Figures 3 and 4). Additionally, Figure 5 substantiated the link between GDM history and UL through elevated BMI levels. After adjusting for age, race, education level, poverty income ratio, and smoking status, a nonlinear positive correlation emerged between BMI and the risk of UL in individuals with a GDM history. Furthermore, a threshold effect analysis pinpointed a critical inflection point at a BMI value of 34.88.

### 3.5 The Relationship Between UL Grading and Variables

Table 4 highlights a significant and intricate correlation between age, BMI, white blood cell count WBC, and SII concerning the severity of UL. The results suggest that older women with higher BMI, elevated white blood cell counts, and increased SII levels are more likely to experience more severe forms of UL. This finding underscores the intricate interplay of these health indicators and their impact on women's health.

**Table 4:** Relationship between variables and severity of UL.

Variable names	Overall	Never UL	UL 1	UL 2	UL 3	UL 4	*p*
*n*	13,408	7196	1834	1886	1157	1335	
Age	52.2 ± 16.61	48.89 ± 16.62	51.99 ± 15.42	54.58 ± 15.3	56.96 ± 15.71	62.77 ± 14.56	< 0.01
BMI (kg/m2)	30.16 ± 7.63	29.13 ± 7.22	30.1 ± 7.42	30.92 ± 7.58	31.99 ± 7.8	33.2 ± 8.72	< 0.01
Normal weight (%)	3544 (26.43)	2256 (31.35)	496 (27.04)	397 (21.05)	196 (16.94)	199 (14.91)	< 0.01
Overweight (%)	3918 (29.22)	2171 (30.17)	520 (28.35)	548 (29.06)	324 (28.00)	355 (26.59)	

## 4 Discussion

GDM is characterized by glucose intolerance that develops during pregnancy, affecting an estimated 14% of pregnant women worldwide [1, 2]. This condition presents considerable immediate and prolonged health risks for both the mother and the fetus, including complications such as macrosomia, obesity, and various metabolic disorders [3]. As the global prevalence of GDM continues to increase, its impact on maternal health and quality of life is becoming more pronounced, underscoring the urgent need to address its potential effects on women's comprehensive health and daily functioning [9, 10, 11].

The severity arises from various factors, including age, BMI, WBC, and SII, all of which play significant roles [12, 13, 14, 15]. Evidence suggests that older women or higher BMI often present with more severe UL, potentially due to hormonal changes and changes in muscle mass associated with aging and an increased risk of intra‐abdominal pressure, which can intensify both the symptoms and severity of UL [17, 18, 19].

Moreover, an increase in WBC counts may serve as an indicator of chronic inflammation, which has been implicated in the development of various gynecological disorders [20, 21]. These disorders can further affect intra‐abdominal pressure. A higher WBC may suggest a more significant inflammatory state, which may associated with the occurrence and development of UL. This finding underscore the importance of considering these clinical parameters in the management and treatment of UL. Targeted therapies aimed at modulating inflammation may offer new avenues for intervention, particularly for women exhibiting these risk factors. Future research should aim to elucidate the underlying mechanisms connecting this variable to UL severity, potentially leading to more personalized treatment strategies.

The finding of this study reveal a correlation between a history of GDM and the risk of UL in women, particularly in those who have a history of GDM with smoking status. Polycystic ovary syndrome (PCOS) is a common endocrine disorder in women of reproductive age. Research indicates that patients with PCOS have a higher risk of developing GDM during pregnancy. These patients may have a risk of UL due to elevated testosterone levels or obesity [22, 23]. These results underscore the necessity for clinical practices to focus on the thorough management of women with a history of GDM, integrating lifestyle factors, particularly smoking behaviors, into the key content of assessment and intervention, which can help improve their quality of life. Additionally, it also points to future research directions, exploring the mechanisms by which smoking exacerbates the risk of UL in women with GDM and identifying effective intervention points.

Moreover, our study identified a critical inflection point in the relationship between BMI and the risk of UL among women with a history of GDM. Specifically, a nonlinear positive association was observed, with the inflection point occurring at a BMI of 34.88. This value, which lies within the Class I obesity category (BMI 30–34.9) and near the threshold for Class II obesity (BMI 35–39.9), suggests that the risk of UL may escalate more rapidly once BMI exceeds this level. Clinically, this finding highlights the importance of weight management in women with a history of GDM, particularly for those whose BMI approaches or surpasses 34.88. Given that obesity is a modifiable risk factor, interventions aimed at preventing BMI from exceeding this threshold could be crucial in reducing the risk of UL. This is further supported by our mediation analysis, which demonstrated that BMI significantly mediates the association between GDM and UL, accounting for 53.4% of the relationship. Therefore, targeted weight management strategies should be considered an integral component of care for women with a history of GDM to mitigate their risk of developing UL.

The present investigation into the correlation between GDM and female UL utilizing data from the NHANES database. It provides a novel area of exploration in the field of reproductive health. It notably elucidates the mediating effects of BMI and WBC counts in this relationship, thereby addressing a significant void in the current body of literature. Previous research has established connections between obesity and urinary dysfunction [24, 25], as well as between inflammation and metabolic disorders [26, 27], but our findings uniquely indicate that both BMI and WBC counts are not only correlated with GDM but also exacerbate the risk of UL in affected women. Metformin is generally regarded as a first‐line therapeutic agent for patients with GDM. Studies suggest that metformin reduces the risk of GDM by promoting weight loss and ameliorating metabolic disturbances, with weight management established as a critical intervention for UL [28]. This mechanism may partially account for the mediating effect of BMI observed in this study. Notably, patients with PCOS, a high‐risk group for GDM, are often managed with supplements such as inositol and vitamin D, alongside metformin. Existing research indicates that these interventions may indirectly mitigate the risk of GDM‐related complications by suppressing systemic inflammation. However, the association between vitamin D deficiency and pelvic floor muscle dysfunction suggests that nutritional status may further influence the incidence of UL [29]. Future studies are needed to clarify whether these interventions might exacerbate or alleviate the risk of UL in GDM patients.

Our findings have significant implications for clinical practice. At present, the diagnosis and evaluation of UL predominantly rely on symptoms, questionnaires, muscle strength assessments, and various imaging techniques [30, 31]. The identification of mediating variables linking gestational diabetes mellitus (GDM) with UL could improve diagnostic tests and assist in identifying potential objective indicators. Furthermore, these results indicate that healthcare providers should prioritize these factors when evaluating the risk of urinary dysfunction in pregnant women with GDM. Integrating early weight management and monitoring of inflammatory markers into a personalized, comprehensive management strategy for GDM patients could substantially enhance their quality of life.

To the best of our knowledge, this is the first study to explore the potential relationship between GDM and UI, revealing that BMI and WBC count may modify their effects. However, our study has several limitations. As it relies on the NHANES database, the cross‐sectional design complicates establishing causality, and the generalizability of our findings may be limited since the database includes only the U.S. population. Additionally, the self‐reported nature of exposure and outcome variables introduces the potential for self‐report and recall biases, which could overlook asymptomatic cases of UL. Furthermore, the database lacks data on certain UL risk factors, such as the duration of the second stage of labor and newborn birth weight, potentially rendering our adjustments insufficiently comprehensive. Moreover, the cross‐sectional approach restricts our ability to understand how changes in BMI and WBC over time influence this relationship. Future research should prioritize longitudinal designs and objective measurements to strengthen the validity of conclusions drawn from this study.

## Author Contributions

**Huiwen Hu:** conceptualization, writing – original draft. **Bin Yu:** methodology, data curation, validation. **Mei Xiang:** investigation. **Ziyi Guo:** writing – original draft. **Huihui Wang:** supervision, visualization. **Li Wang:** writing – review and editing, funding acquisition.

## Conflicts of Interest

The authors declare no conflicts of interest.

## Transparency Statement

The corresponding authors, Huihui Wang and Li Wang, affirm that this manuscript is an honest, accurate, and transparent account of the study being reported; that no important aspects of the study have been omitted; and that any discrepancies from the study as planned (and, if relevant, registered) have been explained.

## Data Availability

The data from this manuscript can be found in the NHANES section of the CDC website. Data in this manuscript are publicly and freely available without restriction at: https://wwwn.cdc.gov/nchs/nhanes/continuousnhanes/default.aspx?cycle=2017-2020. For any further inquiries, please contact the corresponding author.

## References

[1] E. M. Alfadhli , “Gestational Diabetes Mellitus,” Saudi Medical Journal 36, no. 4 (2015): 399–406. 25828275 10.15537/smj.2015.4.10307 PMC4404472

[2] H. Wang , N. Li , T. Chivese , et al., “IDF Diabetes Atlas: Estimation of Global and Regional Gestational Diabetes Mellitus Prevalence for 2021 by International Association of Diabetes in Pregnancy Study Group's Criteria,” Diabetes Research and Clinical Practice 183 (2022): 109050. 34883186 10.1016/j.diabres.2021.109050

[3] A. Sweeting , J. Wong , H. R. Murphy , and G. P. Ross , “A Clinical Update on Gestational Diabetes Mellitus,” Endocrine Reviews 43, no. 5 (2022): 763–793. 35041752 10.1210/endrev/bnac003 PMC9512153

[4] J. Juan and H. Yang , “Prevalence, Prevention, and Lifestyle Intervention of Gestational Diabetes Mellitus in China,” International Journal of Environmental Research and Public Health 17, no. 24 (2020): 9517. 33353136 10.3390/ijerph17249517 PMC7766930

[5] M. E. Bianco and J. L. Josefson , “Hyperglycemia During Pregnancy and Long‐Term Offspring Outcomes,” Current Diabetes Reports 19, no. 12 (2019): 143. 31754898 10.1007/s11892-019-1267-6 PMC7008468

[6] B. Ugwudike and M. Kwok , “Update on Gestational Diabetes and Adverse Pregnancy Outcomes,” Current Opinion in Obstetrics & Gynecology 35, no. 5 (2023): 453–459. 37560815 10.1097/GCO.0000000000000901

[7] Y. X. Ye , Y. Wang , P. Wu , et al., “Blood Cell Parameters From Early to Middle Pregnancy and Risk of Gestational Diabetes Mellitus,” Journal of Clinical Endocrinology & Metabolism 108, no. 12 (2023): e1702–e1711. 37279929 10.1210/clinem/dgad336

[8] J. Quaghebeur , P. Petros , J. J. Wyndaele , and S. De Wachter , “Pelvic‐Floor Function, Dysfunction, and Treatment,” European Journal of Obstetrics & Gynecology and Reproductive Biology 265 (2021): 143–149. 34492609 10.1016/j.ejogrb.2021.08.026

[9] W. R. Grimes and M. Stratton , “Pelvic Floor Dysfunction,” in StatPearls [Internet] (StatPearls Publishing, 2023). 32644672

[10] D. H. Thom and G. Rortveit , “Prevalence of Postpartum Urinary Incontinence: A Systematic Review,” Acta Obstetricia et Gynecologica Scandinavica 89, no. 12 (2010): 1511–1522. 21050146 10.3109/00016349.2010.526188

[11] S. Åhlund , E. Rothstein , I. Rådestad , S. Zwedberg , and H. Lindgren , “Urinary Incontinence After Uncomplicated Spontaneous Vaginal Birth in Primiparous Women During the First Year After Birth,” International Urogynecology Journal 31, no. 7 (2020): 1409–1416. 31139858 10.1007/s00192-019-03975-0 PMC7306031

[12] M. Datar , L. C. Pan , J. L. McKinney , T. F. Goss , and S. J. Pulliam , “Healthcare Resource Use and Cost Burden of Urinary Incontinence to United States Payers,” Neurourology and Urodynamics 41, no. 7 (2022): 1553–1562. 35708134 10.1002/nau.24989 PMC9542745

[13] N. Kupfer , A. Clancy , F. Maguire , and J. Stairs , “Prevalence and Risk Factors for Urinary Incontinence in Nulliparous Women: A Contemporary, Population‐Based Cohort Study,” Urogynecology 29, no. 5 (2023): 520–527. 36730707 10.1097/SPV.0000000000001296

[14] K. Wang , X. Xu , G. Jia , and H. Jiang , “Risk Factors for Postpartum Stress Urinary Incontinence: A Systematic Review and Meta‐Analysis,” Reproductive Sciences 27, no. 12 (2020): 2129–2145. 32638282 10.1007/s43032-020-00254-y

[15] H. F. A. Moossdorff‐Steinhauser , B. C. M. Berghmans , M. E. A. Spaanderman , and E. M. J. Bols , “Prevalence, Incidence and Bothersomeness of Urinary Incontinence in Pregnancy: A Systematic Review and Meta‐Analysis,” International Urogynecology Journal 32, no. 7 (2021): 1633–1652. 33439277 10.1007/s00192-020-04636-3 PMC8295103

[16] R. M. Baron and D. A. Kenny , “The Moderator‐Mediator Variable Distinction in Social Psychological Research: Conceptual, Strategic, and Statistical Considerations,” Journal of Personality and Social Psychology 51, no. 6 (1986): 1173–1182. 3806354 10.1037//0022-3514.51.6.1173

[17] W. Liu and L. Qian , “Risk Factors for Postpartum Stress Urinary Incontinence: A Prospective Study,” BMC Urology 24, no. 1 (2024): 42. 38365685 10.1186/s12894-024-01430-x PMC10873983

[18] C. Molinet Coll , E. Martínez Franco , L. Altimira Queral , D. Cuadras , L. Amat Tardiu , and D. Parés , “Hormonal Influence in Stress Urinary Incontinence During Pregnancy and Postpartum,” Reproductive Sciences (Thousand Oaks, Calif.) 29, no. 8 (2022): 2190–2199. 35471548 10.1007/s43032-022-00946-7

[19] S. Iguchi , T. Inoue‐Hirakawa , I. Nojima , T. Noguchi , and H. Sugiura , “Relationships Between Stress Urinary Incontinence and Trunk Muscle Mass or Spinal Alignment in Older Women,” LUTS: Lower Urinary Tract Symptoms 14, no. 1 (2022): 10–16. 34288434 10.1111/luts.12403 PMC9290447

[20] A. Fuselier , J. Hanberry , J. Margaret Lovin , and A. Gomelsky , “Obesity and Stress Urinary Incontinence: Impact on Pathophysiology and Treatment,” Current Urology Reports 19, no. 1 (2018): 10. 29468457 10.1007/s11934-018-0762-7

[21] J. Womack , P. C. Tien , J. Feldman , et al., “Obesity and Immune Cell Counts in Women,” Metabolism: Clinical and Experimental 56, no. 7 (2007): 998–1004. 17570264 10.1016/j.metabol.2007.03.008 PMC1939725

[22] T. Montezuma , F. I. Antônio , A. C. J. de Sá Rosa e Silva , M. F. S. de Sá , R. A. Ferriani , and C. H. J. Ferreira , “Assessment of Symptoms of Urinary Incontinence in Women With Polycystic Ovary Syndrome,” Clinics 66, no. 11 (2011): 1911–1915. 22086521 10.1590/S1807-59322011001100010 PMC3203963

[23] F. I. Antônio , K. Bo , R. A. Ferriani , M. F. S. de Sá , A. C. J. de Sá Rosa e Silva , and C. H. J. Ferreira , “Pelvic Floor Muscle Strength and Urinary Incontinence in Hyperandrogenic Women With Polycystic Ovary Syndrome,” International Urogynecology Journal 24, no. 10 (2013): 1709–1714. 23575700 10.1007/s00192-013-2095-x

[24] H. S. Lee , I. H. Koh , H. S. Kim , and Y. J. Kwon , “Platelet and White Blood Cell Count Are Independently Associated With Sarcopenia: A Nationwide Population‐Based Study,” Thrombosis Research 183 (2019): 36–44. 31614293 10.1016/j.thromres.2019.09.007

[25] C. Chilaka , P. Toozs‐Hobson , and V. Chilaka , “Pelvic Floor Dysfunction and Obesity,” Best Practice & Research Clinical Obstetrics & Gynaecology 90 (2023): 102389. 37541114 10.1016/j.bpobgyn.2023.102389

[26] D. J. Osborn , M. Strain , A. Gomelsky , J. Rothschild , and R. Dmochowski , “Obesity and Female Stress Urinary Incontinence,” Urology 82, no. 4 (2013): 759–763. 23972338 10.1016/j.urology.2013.06.020

[27] L. Khambule and J. A. George , “The Role of Inflammation in the Development of GDM and the Use of Markers of Inflammation in GDM Screening,” Advances in Experimental Medicine and Biology 1134 (2019): 217–242. 30919340 10.1007/978-3-030-12668-1_12

[28] A. B. Arouca , A. M. Santaliestra‐Pasías , L. A. Moreno , et al., “Diet as a Moderator in the Association of Sedentary Behaviors With Inflammatory Biomarkers Among Adolescents in the HELENA Study,” European Journal of Nutrition 58, no. 5 (2019): 2051–2065. 29974229 10.1007/s00394-018-1764-4

[29] D. Menichini , G. Forte , B. Orrù , G. Gullo , V. Unfer , and F. Facchinetti , “The Role of Vitamin D in Metabolic and Reproductive Disturbances of Polycystic Ovary Syndrome: A Narrative Mini‐Review,” International Journal for Vitamin and Nutrition Research 92, no. 2 (2022): 126–133. 33284035 10.1024/0300-9831/a000691

[30] N. P. Joshi , S. D. Madiwale , D. P. Sundrani , and S. R. Joshi , “Fatty Acids, Inflammation and Angiogenesis in Women With Gestational Diabetes Mellitus,” Biochimie 212 (2023): 31–40. 37059350 10.1016/j.biochi.2023.04.005

[31] A. K. Nambiar , R. Bosch , F. Cruz , et al., “EAU Guidelines on Assessment and Nonsurgical Management of Urinary Incontinence,” European Urology 73, no. 4 (2018): 596–609. 29398262 10.1016/j.eururo.2017.12.031

